# Does toe clipping for genotyping interfere with later-in-life nociception in mice?

**DOI:** 10.1097/PR9.0000000000000740

**Published:** 2019-04-25

**Authors:** Noémie Frezel, Gilles Kratzer, Philipp Verzar, Jérôme Bürki, Fabienne A. Weber, Hanns Ulrich Zeilhofer

**Affiliations:** a Institute of Pharmacology and Toxicology, University of Zurich, Zurich, Switzerland; b Institute of Biology (IBENS), École Normale Supérieure, CNRS, INSERM, Paris Sciences et Lettres Research University, Paris, France; c Department of Mathematics, University of Zurich, Zurich, Switzerland; d Laboratory Animal Services Center, Zurich, Switzerland; e Institute of Pharmaceutical Sciences, Swiss Federal Institute of Technology (ETH) Zurich, Zurich, Switzerland

**Keywords:** Mouse, Pain, Genotyping, Priming, Trauma, Nociception, Pain model, Biopsy, Toe clipping, Surgery, Postoperative pain

## Abstract

**Introduction:**

Genetically modified mice are widely used in studies on human and animal physiology and pharmacology, including pain research. The experimental design usually includes comparisons of genetically modified mice with wild-type littermates, requiring biopsy material for genotyping and methods for unequivocal identification of individual mice. Ethical standards and, in some countries, legislation require that both needs are reached with a single procedure. Clipping of the most distal phalanx of up to two toes per paw (toe clipping) is the favored procedure in most research fields, but it may be problematic in sensory physiology and pain research.

**Objectives:**

To systematically investigate whether toe-clipping influences later-in-life nociceptive sensitivity or the susceptibility to neuropathic or inflammatory hyperalgesia.

**Methods:**

We tested in male mice whether the clipping of 2 toes of a hind paw influences nociceptive sensitivities to noxious heat or cold, or to mechanical stimulation under baseline conditions, after peripheral nerve injury (chronic constriction of the sciatic nerve) or during peripheral inflammation induced by subcutaneous zymosan A injection. We tested not only for the presence of significant differences but also specifically addressed bioequivalence using the 2 one-sided t test procedure. We chose a threshold of 25% variation of the control value for nonequivalence, which is usually taken as a threshold for biological relevance in pain tests.

**Results:**

Using this value, we found that for all conditions (non-neuropathic and non-inflamed, neuropathic and inflamed), nociceptive sensitivities significantly fell within the equivalence bounds of the non–toe-clipped control mice.

**Conclusions:**

These results suggest that toe clipping does not have long-term effects on nociceptive sensitivities and does not alter the susceptibility of male mice to neuropathic or inflammatory hyperalgesia.

## 1. Introduction

In many countries, legislation requires that tissue biopsies for genotyping and the marking of the mice are done in a single procedure, meaning that the biopsies must be taken in a way that allows the later identification of the mice without further tagging.^5^ As a consequence, procedures that have been used in the past such as the combination of tail biopsies and application of ear tags are no longer permitted. Alternative procedures that comply with the legal requirements include ear punches after weaning and toe clipping in mice before weaning. Toe clipping, that is, the amputation of the most distal phalanx of 1 to 2 toes, at around 10 days of age is often the preferred technique because it ensures that the results of the genotyping are already available at weaning thus saving costs and space for mouse keeping.^19^ These amputations bear in principle the risk of transient or persistent impairment of sensory functions, potentially confounding the results of subsequent experiments.

Previous studies found no long-term impact of toe clipping on motor behavior and well-being of mice^6,15,19^ but assessed either only heat responses in naive mice^19^ or did not address potential changes in nociception at all.^6,15^ With respect to pain research, we have identified at least 3 important gaps in knowledge. First, the impact of toe-clipping on outcomes of tests typically used in current pain research^8^ has not yet been systematically assessed. Second, acute injury and inflammation can prime rodents to the development of exaggerated inflammatory hyperalgesia upon a second inflammatory insult later in life.^1,3,17^ It is thought that long-lasting changes underlying these priming mechanisms reside in the peripheral nerve endings. It has not yet been tested if toe clipping induces such nociceptive priming. Other work suggests that, in particular, injury or inflammation occurring early in life leads to higher sensitization in chronic pain models induced later in the adult.^18^ Consequently, even if postnatal toe clipping does not alter baseline somatosensory or nociceptive responses, we considered that it might still predispose mice to stronger inflammatory or neuropathic hyperalgesia later in life.^13,17,18^ Third, previous studies concluded on the basis of the absence of a statistically significant difference that toe clipping would not affect sensory sensitivity. However, the absence of a significant difference between experimental groups does not mean that these groups can be considered equivalent, in a statistical perspective.^10^

In the present study we have addressed these 3 gaps in knowledge. We not only provide a detailed analysis of the sensitivity of toe-clipped mice to acute nociceptive stimulation but also compare the susceptibility of toe-clipped (TC) and non-TC control mice to neuropathic and inflammatory hyperalgesia. Furthermore, in all these assays, we performed a bioequivalence test using the two one-sided *t* test (TOST) procedure.^10^ Our results show that within a reasonable boundary of ±25% (compare Ref. 7), toe-clipped mice neither do exhibit altered somatosensory or pain sensitivity nor do they differ from non-TC mice in their susceptibility to neuropathic or inflammatory hyperalgesia.

## 2. Methods

### 2.1. Animals and toe-clipping procedure

C57BL/6J mice were maintained under a 24 hours light–dark cycle with ad libitum access to food and water. Pups were weaned at postnatal day 21. Toe clipping was performed by trained animal caretakers. The clipping of 2 toes from one hind paw corresponds the usual maximum of toe-clipping–induced tissue injury. According to the numbering system commonly used to identify the mice with toe clipping,^15^ clipping of no more than 2 toes in one hind paw already allows marking of 19 mice, and up to 99 including the forepaws, which is sufficient even for a cage with 2 litters. Partial amputations in the second phalange of the toes were done in neonatal male pups at the age of 10 days. For the clipping procedure, the entire litter was taken out of the nest and put into an extra cage lid laid out with paper tissue. The sex of all animals was checked, and the male pups of the litter were divided into 2 groups: 50% of the males were used as control animals, which underwent no clipping procedure. The other half underwent the clipping procedure by being held in hand over a paper pad and, with gentle pressure, restrained. The second phalanges of the third and fifth toes of the left hind limb (counted from the most inner phalange) were cut. Between each animal, the instrument was wiped with alcohol. After the complete group had been processed, the animals were put back to the mother in the home cage nest. All mice were also marked by ear punch performed at weaning in order to identify individuals for the experiments. Permission has been obtained from the veterinary office of the canton Zürich (license number 063/2016).

### 2.2. Behavioral responses to nociceptive stimulation

Mice were randomly assigned to TC or non-TC (Cont., control) groups, and all behavioral tests were performed by an experimenter blinded to the group of the mice. The term “naive mice” refers here to adult (7–8 weeks old) mice that were not subjected to chronic constriction of the left sciatic nerve (chronic constriction injury [CCI]) or zymosan A injection. Only 1 test was performed per day and mouse.

#### 2.2.1. Mechanical sensitivity

Mice were placed in Plexiglas chambers (8 × 8 cm) on a raised wire grid and allowed to acclimatize for at least 1 hour before testing. Withdrawal thresholds where assessed by the stimulation of the hind paw with an electronic von Frey anesthesiometer (IITC, Woodland Hills, CA). Eight measurements are taken at an interval of 10 minutes.

Sensitivity to light touch or acute painful stimulation was also tested. Both hind paws were stimulated alternately, and 10 measurements were taken of each hind paw. For light touch, mice were gently touched (from the bottom of the grid) with a soft paint brush on the plantar surface of the hind paw. For acute painful stimulation, the plantar surface of hind paws was stimulated with a blunted G26 needle without penetration of the skin. For both tests, each response to this stimulation was quantified by a score of 0 or 1 (no evoked movement = 0, walking away or brief paw lifting for ≤1 second = 1) and plotted as a percentage of positive responses (ie, a mouse that responded 8 of 10 times [score 1] yielded an average score of 80%).

#### 2.2.2. Cold sensitivity

Mice were placed in Plexiglas chambers (8 × 8 cm) on a 5-mm-thick borosilicate glass platform and allowed to acclimatize for at least 1 hour before testing. A dry ice pellet was applied to the surface of the glass below the paw, cooling the surface. Withdrawal thresholds were measured using a stopwatch, and a cutoff time of 20 seconds was set.

#### 2.2.3. Heat sensitivity (Hargreaves test)

Mice were placed in Plexiglas chambers (8 × 8 cm) on a glass surface and allowed to acclimatize for at least 1 hour before testing. A movable infrared generator was placed below the plantar surface of one hind paw. Withdrawal thresholds were recorded automatically by an electronically controlled commercially available instrument with a built-in timer (Plantar Analgesia Meter; IITC, Woodland Hills, CA), and a cutoff time of 32 seconds was set. Eight measurements were taken at an interval of 10 minutes.

#### 2.2.4. Motor coordination (rotarod)

Mice were placed onto a rotarod setup (IITC). The rod was set to accelerate from 4 to 40 rpm over a period of 300 seconds. Two training sessions were performed before the latency to fall was measured in 5 test sessions per mouse.

#### 2.2.5. Chronic pain models

Neuropathic pain was studied using the CCI model. Seven- to 8-week-old TC mice and non-TC control mice underwent constriction injury of the left sciatic nerve just proximal to the trifurcation was performed as described previously.^4^ Anesthesia was induced and maintained by 2% isoflurane (Provet AG, Lyssach, Switzerland), combined with oxygen (30%). Before the start of the surgery, mice received 0.2 mg/kg buprenorphine subcutaneously. The sciatic nerve was exposed at the midthigh level proximal to the sciatic trifurcation by blunt dissection through the *biceps femoris*. Three chromic gut ligatures (5/0) were tied loosely around the nerve with about 1-mm spacing. The ligatures were tied until they elicited a brief twitch in the hind limb. The incision was closed in layers. Inflammatory pain was studied in the zymosan A model. Under short anesthesia, zymosan A (Sigma-Aldrich, St Louis, MO, 0.06 mg in 20 µL NaCl) was injected subcutaneously into the plantar side of the left hind paw.

### 2.3. Statistical analysis

All data are presented as boxplots. Statistical analysis was performed as follows: group means of TC and Cont. naive mice for all behavioral tests were compared using a 2-sided unpaired Student *t* test, assuming equal variance. For the CCI and zymosan A groups, means were compared using a 2-way repeated measures analysis of variance (ANOVA), followed by pairwise comparisons with Sidak adjustment for multiple comparisons (*t* tests and ANOVA performed with SPSS: IBM Corp. Released 2017. IBM SPSS Statistics for Windows, Version 25.0. Armonk, NY). In all cases, bioequivalence was tested using the TOST procedure (Two One-Sided T-tests^10^ using R and TOSTER library^16^). Behavioral responses are reported as mean ± SEM.

## 3. Results

### 3.1. Somatosensory and nociceptive sensitivity in naive toe-clipped mice

**Figure 1.**
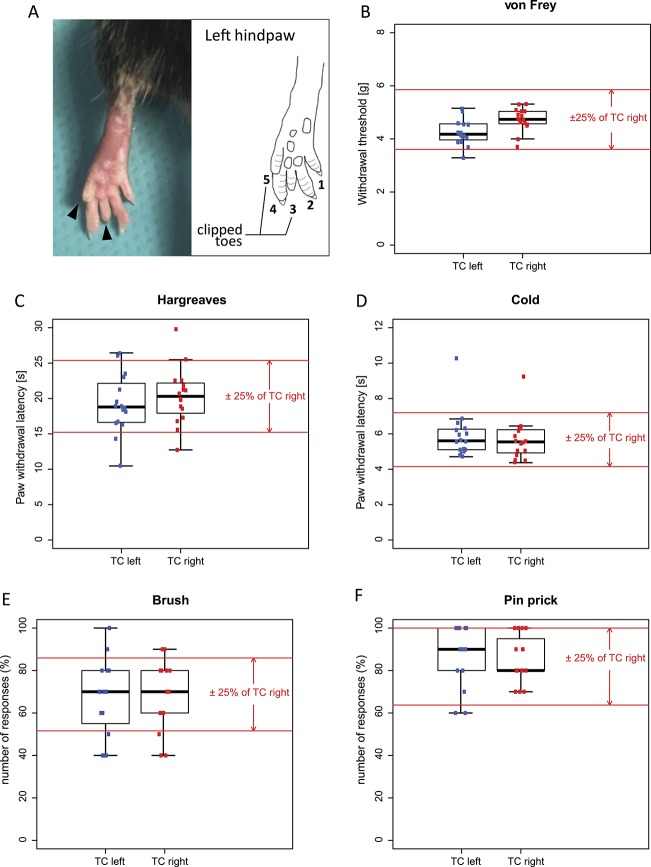
Somatosensory and nociceptive sensitivity in naive toe-clipped mice. (A) Left hind paw of an adult mouse. Toes “3” and “5” have been clipped at the age of 10 days. (B–F) For all tests, mean response values of the left paws were significantly within the equivalence bounds of ±25% of the values of the right paw (red lines, Table 1). (B) There is a statistically significant but very small difference in sensitivity to mechanical stimuli between the left and right hind paws. Clipping had no significant effect on heat (C), cold (D), light touch (E), or nociceptive (F) sensitivity. TC left, n = 16; TC right, n = 16. TC, toe clipped; Cont, control. For details of the statistical analyses, Table 1. TC, toe clipped.

To test if toe clipping had an effect on acute somatosensory or nociceptive sensitivity, we investigated mice that had undergone toe clipping (2 tiptoes of the left hind paw) at the age of 10 days (Fig. 1A). Responses of the left (TC) and right (non-TC, control) paws were compared about 6 weeks after toe clipping (ie, at an age of 6 to 8 weeks). A battery of somatosensory or nociceptive tests was applied, including the von Frey and brush tests (for static and dynamic mechanical sensitivity), pinprick test (for noxious mechanical sensitivity), the Hargreaves test (for noxious heat sensitivity), and dry ice test (for cold sensitivity).

In none of these tests, except the von Frey test, we detected statistically significant differences in responses upon stimulation of the left or right hind paw with *P* values (unpaired Student *t* test) between 0.45 and 0.75 (Fig. 1B–F, unpaired Student *t* test, Table 1). A statistically significant difference (*P* = 0.0095, unpaired Student *t* test) was found in the withdrawal thresholds after von Frey stimulation, but this difference was very small (mean of 4.29 ± 0.12 vs 4.72 ± 0.11, for left and right hind paws, respectively). To examine whether the responses were similar (equivalent) within certain boundaries, we performed a TOST procedure.^10^ Mean response values of the left paws were significantly within the equivalent bounds of ±25% of the values of the right (control) paws (Fig. 1 and Table 1). Smaller differences are unlikely to be meaningful biologically.^7,14^

**Table 1:**
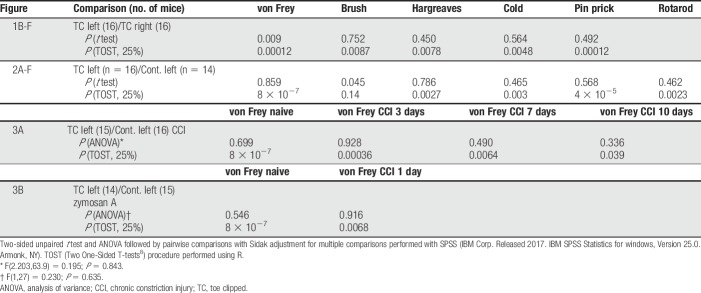
*P* values for all comparisons in Figures 1–3.

**Figure 2.**
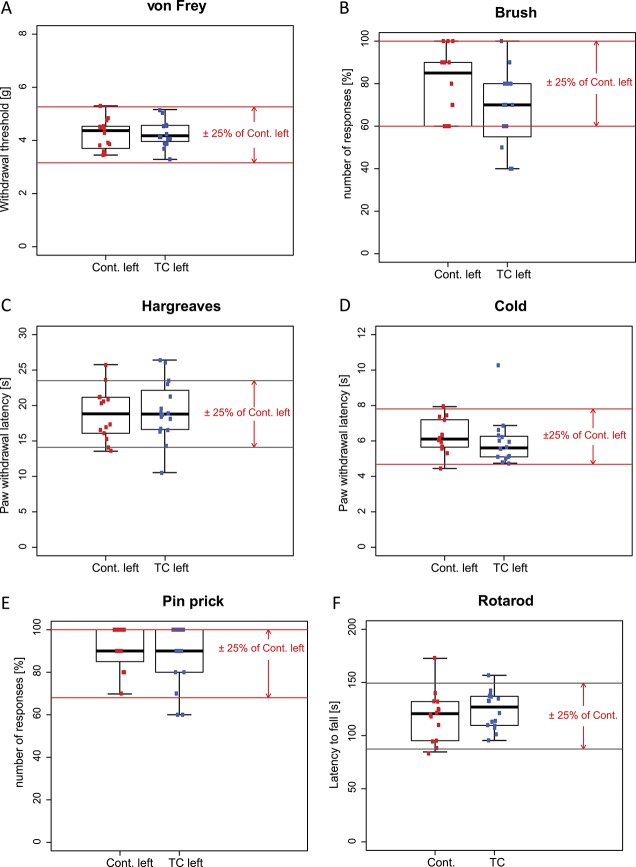
Somatosensory and nociceptive sensitivity in naive toe-clipped mice compared with control mice. Response thresholds to mechanical stimulation with von Frey filaments (A), response scores to light mechanical brush stimulation (B), withdrawal thresholds to noxious heat (C) and noxious cold (D) stimulation, and response scores to noxious mechanical stimulation (E). (F) Rotarod performance. For all tests, except brush stimulation, mean response values of the left paws were significantly within the equivalence bounds of ±25% of the values of the right paw (red lines). TC left, n = 16; Cont. left, n = 14. TC, toe clipped; Cont, control. For details of the statistical analyses, Table 1. TC, toe clipped.

The absence of a sensory difference between TC and non-TC paws of the same mouse excludes local sensitizing effects of toe clipping but leaves the possibility open that toe clipping might induce systemic (bilateral) effects. We therefore compared the left hind paws of non-TC control mice (Cont.) and TC mice (Fig. 2 A-E). All *P* values except one fell between 0.46 and 0.86 (unpaired Student *t* test, Table 1). A statistically significant difference was only detected for the brush test (*P* = 0.045, unpaired Student *t* test). The subsequent TOST procedure indicated that, for all tests, except the brush test, response scores of the TC mice were significantly within the equivalent bounds of ±25% of the control group (Table 1). We also tested performance in the rotarod test to assess general motor performance and found no significant differences between TC and control mice (Fig. 2F and Table 1).

### 3.2. Susceptibility of toe-clipped mice to neuropathic and inflammatory hyperalgesia

The absence of altered somatosensory and pain sensitivity in TC mice does not exclude changes in the nociceptive system that manifest only later in life under certain conditions. For example, it has been reported that inflammatory insults can “prime” the nociceptive system leading to prolonged or exaggerated hyperalgesia upon exposure to a second inflammatory event later in life.^9,13,17,18^ We have therefore included an analysis of the susceptibility of adult TC mice to inflammatory and neuropathic hyperalgesia in our study. We induced neuropathic pain using the CCI model and inflammatory pain by injecting zymosan A into the plantar surface of the left hind paw and assessed hypersensitivity to punctate mechanical stimuli using the von Frey test.

**Figure 3.**
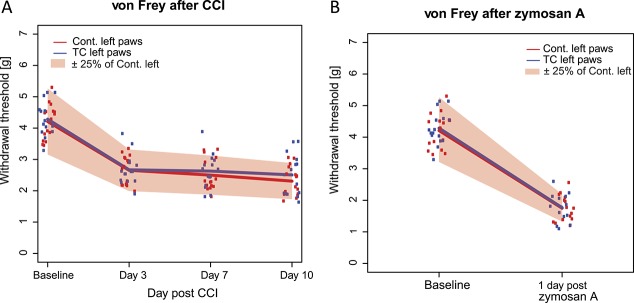
Susceptibility of toe-clipped mice to neuropathic and inflammatory hyperalgesia. For both tests, mean response values of the left paws were significantly within the equivalence bounds of ±25% of the values of the right paw (pink shaded area, Table 1). (A) Withdrawal thresholds of the left hind paws decreased at day 3 after the surgery and maintained a steady-state level of sensitization at days 7 and 10 after the surgery in both TC and control mice. TC, n = 15; Cont, n = 16. (B) Withdrawal thresholds of the left hind paws decreased at day 1 after injection of zymosan A in both TC and control mice. TC, n = 14; Cont., n = 15. TC, toe -clipped; Cont, control. For details of the statistical analyses, Table 1.

As expected, after CCI of the left sciatic nerve, the withdrawal thresholds of the left hind paws decreased at day 3 after the surgery and maintained a steady-state level of sensitization at days 7 and 10 after the surgery (Fig. 3A). At days 3, 7, and 10 after the surgery, we detected no significant differences between responses of the left hind paws of TC and Cont. mice with *P* values between 0.34 and 0.93 (Fig. 3A, F(2.203,63.9) = 0.195; *P* = 0.843, ANOVA followed by pairwise comparisons with Sidak adjustment for multiple comparisons, for more details Table 1). Two one-sided *t* test procedures demonstrated that mean responses of the TC mice significantly fell within ±25% equivalence bounds of the mean response scores of the Cont. mice for each day (with *P* = 0.00036, *P* = 0.0064, and *P* = 0.039, at days 3, 7, and 10, respectively, Table 1).

Finally, TC and Cont. mice were injected with zymosan A in the plantar surface of the left hind paw. One day after injection, mice developed a strong hypersensitivity to von Frey stimulation. We detected no significant differences between the responses of the left hind paws of TC and Cont. mice (Fig. 3B, F(1,27) = 0.230; *P* = 0.635, ANOVA followed by pairwise comparisons with Sidak adjustment for multiple comparisons, for more details Table 1). Furthermore, the response values of the TC left paws, both at baseline and 1 day after injection, significantly fell within the equivalent bounds of ±25% of the values for the controls (*P* = 0.0068 at day 1; Table 1).

## 4. Discussion

The widespread use of genetically modified mouse lines in many areas of research requires appropriate procedures to collect tissue samples for genotyping and identification of the individual mice. Toe clipping of pups is one of the few procedures that comply with European legislation.^5^ In addition, it allows to take biopsies and to unambiguously mark mice within their litter in a single procedure early after birth, thus saving space and financial resources.^5,19^ Some concerns have however been raised, specifically in the field of pain research, because many of the assays commonly used rely on the sensory stimulation of the hind paws of the mouse.^8^ It is therefore essential to ensure that the tissue sampling procedure does not interfere with the sensitivity or responsiveness of the mice in these assays. Previous studies have addressed the general well-being of the mice both around the time of clipping and later in life.^6,15,19^ One study that addressed acute nociception showed that the response to the hot plate test was not affected by toe clipping.^19^ However, the potential effect on von Frey, cold and hot plantar assays, sensitivity to light touch or pinprick, or the impact on the development of hypersensitivity during chronic pain models remained unknown.

Here, we analyzed the impact of toe clipping, done at 10 days of age, on the somatosensory and nociceptive sensitivity later in life. We show that this procedure left acute somatosensory and nociceptive thresholds measured at the age of 7 to 8 weeks unchanged. The absence of significant changes was verified both for the TC and non-TC paws of the same mouse and also for the comparison between the left hind paws of TC and non-TC mice. The latter comparison is particularly important because previous work has found systemic (bilateral) effects caused by increases in the opioid tone.^12^ Our study failed to reveal an impact of toe clipping on the later-in-life susceptibility to inflammatory or neuropathic hyperalgesia. In one case (von Frey test, Fig. 1B), we observed a statistically significant difference, but the effect size was very small casting doubts on the biological relevance of this result. It is well established that noxious insults early in life change acute nociceptive sensitivity later in life and also lead to so-called hyperalgesic priming.^9,13,17^ Several studies specifically investigated the consequences of perinatal or early postnatal inflammation or injury on later-in-life nociception. These studies revealed that rodents that had undergone perinatal inflammation induced with carrageenan^11,12^ or complete Freund's adjuvant^18^ exhibited reduced acute nociception later in life. Interestingly, the same animals showed stronger hyperalgesia in response to a second inflammatory challenge. Our results show that mild-to-moderate tissue trauma, even when it occurs early in life, does not necessarily lead to long-lasting or persistent changes in nociception or increased susceptibility to inflammatory or neuropathic hyperalgesia. Previous studies revealed critical age windows during which animals were particularly susceptible to the development of long-term changes in nociception. In rats, this critical age window was between birth and postnatal day 8.^11^ It is unknown whether the same time window also applies to mice, but our results, which failed to detect a significant impact on nociception of toe clipping on postnatal day 10, are in line with these previous data. Interestingly, the previous studies found that the impact on later-in-life nociception was stronger in female rats than in male rats.^11^ This gender difference may also contribute to the negative findings in our study.

To the best of our knowledge, previous studies addressing a potential impact of toe clipping on somatosensation and nociception have only tested for the presence of statistically significant differences. However, the absence of such differences does not mean that TC and control mice exhibit unaltered responses. In more general terms, in a statistical analysis, failing to reject the null hypothesis must not be interpreted as equivalent to accepting the null hypothesis.^10^ To test for the equivalence between TC and control mice or paws, we used the TOST procedure. Using this test, we were able to demonstrate equivalence within boundaries of ±25% of the controls for all nociceptive tests. In only 1 case (brush test, Fig. 2B), statistical significance of equivalence was not reached, but even there, the effect size was small, indicating questionable biological relevance. The results of the TOST, of course, depend on the predefined boundaries. For our analyses, we have chosen ±25% of the respective average control values. This value was based on the implicit assumption that differences smaller than 25% are unlikely to be of biological relevance in mouse pain studies. The choice of this threshold is supported by general recommendations made for clinical studies in humans, which consider a reduction in pain intensity of 20% to 30% after treatment as minimal to moderate.^2,7,14^

In summary, our results suggest that toe clipping does neither change somatosensory or nociceptive thresholds and does not predispose to higher-than-normal hyperalgesia in inflammatory or neuropathic pain models. Toe clipping can therefore safely be applied in mice bread for somatosensory or pain research.

## Disclosures

The authors declare that they have no conflict of interest.
